# Recombination-Independent Recognition of DNA Homology for Repeat-Induced Point Mutation (RIP) Is Modulated by the Underlying Nucleotide Sequence

**DOI:** 10.1371/journal.pgen.1006015

**Published:** 2016-05-05

**Authors:** Eugene Gladyshev, Nancy Kleckner

**Affiliations:** Department of Molecular and Cellular Biology, Harvard University, Cambridge, Massachusetts, United States of America; National Cancer Institute, UNITED STATES

## Abstract

Haploid germline nuclei of many filamentous fungi have the capacity to detect homologous nucleotide sequences present on the same or different chromosomes. Once recognized, such sequences can undergo cytosine methylation or cytosine-to-thymine mutation specifically over the extent of shared homology. In *Neurospora crassa* this process is known as Repeat-Induced Point mutation (RIP). Previously, we showed that RIP did not require MEI-3, the only RecA homolog in Neurospora, and that it could detect homologous trinucleotides interspersed with a matching periodicity of 11 or 12 base-pairs along participating chromosomal segments. This pattern was consistent with a mechanism of homology recognition that involved direct interactions between co-aligned double-stranded (ds) DNA molecules, where sequence-specific dsDNA/dsDNA contacts could be established using no more than one triplet per turn. In the present study we have further explored the DNA sequence requirements for RIP. In our previous work, interspersed homologies were always examined in the context of a relatively long adjoining region of perfect homology. Using a new repeat system lacking this strong interaction, we now show that interspersed homologies with overall sequence identity of only 36% can be efficiently detected by RIP in the absence of any perfect homology. Furthermore, in this new system, where the total amount of homology is near the critical threshold required for RIP, the nucleotide composition of participating DNA molecules is identified as an important factor. Our results specifically pinpoint the triplet 5'-GAC-3' as a particularly efficient unit of homology recognition. Finally, we present experimental evidence that the process of homology sensing can be uncoupled from the downstream mutation. Taken together, our results advance the notion that sequence information can be compared directly between double-stranded DNA molecules during RIP and, potentially, in other processes where homologous pairing of intact DNA molecules is observed.

## Author Summary

Recombination-independent pairing of homologous double-stranded DNA molecules is associated with many important biological processes including the alignment of homologous chromosomes in early meiosis, monoallelic gene expression in mammals, and somatic pairing of homologous chromosomes in Drosophila. The molecular mechanism(s) by which homologous sequences are recognized in all of these cases remain(s) largely unknown. The phenomenon of Repeat-Induced Point mutation (RIP) in the filamentous fungus *Neurospora crassa* provides an especially advantageous model system for elucidating the general mechanism of recombination-independent recognition of DNA homology. Here we show that imperfect (interspersed) homologies with overall sequence identity of only 36% can be efficiently detected by RIP in the absence of nearby perfect homology. Under these particularly stringent conditions, specific DNA sequence motifs are found to play a critical role. Our analysis of one such situation identifies the triplet 5'-GAC-3' as an especially favorable unit of homology. We also present experimental evidence that the process of homology recognition for RIP integrates sequence information over hundreds of base-pairs of chromosomal DNA, and that this process can be uncoupled from the downstream mutation. Taken together, our results advance the notion that sequence information can be compared directly between double-stranded DNA molecules during RIP and, potentially, in other processes where homologous pairing of intact DNA molecules is observed.

## Introduction

Many biological systems exhibit specific co-localization (pairing) of homologous DNA molecules independent of recombination-based mechanisms. A paradigmatic example is provided by the persistent association of homologous chromosomes in somatic nuclei of the Diptera insects [1]. Somatic pairing of homologous loci has been documented in yeast (references in [2]) and in mammals (references in [3,4]). In early meiosis, significant pairing of homologous chromosomes occurs prior to or in the absence of recombination in mice, flies, worms, fission yeast and filamentous fungi (references in [2]). The molecular mechanisms by which homologous sequences are recognized in all of these cases remain largely unknown [5]. Roles of direct interactions between DNA molecules or the indirect readout of the base-pair sequence by RNA or proteins have been proposed [6,7].

In addition to the physical co-localization, recombination-independent interactions between homologous DNA molecules can trigger their chemical modification. In filamentous fungi, two closely-related processes can detect duplicated DNA sequences irrespectively of their origin and location in the genome [8]. Once detected, DNA duplications can undergo cytosine methylation (the phenomenon of Methylation Induced Premeiotically, MIP, discovered in *Ascobolus immersu*s [9]) or cytosine-to-thymine mutation (the phenomenon of Repeat-Induced Point mutation, RIP, discovered in *Neurospora crassa* [10]). Both RIP and MIP are restricted to the sexual phase of the fungal life-cycle and occur in haploid germline nuclei that prepare to enter meiosis. Both processes are mediated by specialized cytosine methyltransferases of the RID/Masc1 family [11,12]. Although RIP coincides with a period of increased intrachromosomal recombination [13], it does not require MEI-3, the only RecA homolog in Neurospora [14]. No DNA sequence, with the exception of ribosomal DNA in the nucleolar organizer region, can escape RIP [15]. The general and efficient nature of RIP makes it an especially attractive experimental system for elucidating the general mechanism of recombination-independent recognition of DNA homology.

Previously, we developed a sensitive genetic assay for RIP based on the quantitative analysis of individual mutations induced by a pair of closely-positioned DNA repeats [14]. Using this experimental system, we demonstrated that as few as 155 base-pairs of perfect homology could trigger RIP, and that 220 base-pairs of perfect homology could promote substantial RIP. We further found that the number of RIP mutations accurately reflected the length of homology for repeats ranging between 220 and 520 base-pairs. We then explored the ability of RIP to recognize different patterns of imperfect homology by extending the 220-bp region of perfect homology with additional 200 base-pairs of synthetic DNA that provided partial homology in the adjacent region. Here we discovered that RIP could detect weak partial homologies organized as arrays of base-pair triplets interspersed with a matching periodicity of 11 or 12 base-pairs along participating chromosomal segments. These and other observations led us to propose that sequence homology could be sensed by direct interactions between intact, slightly underwound double-stranded DNA molecules [14].

Our previous studies examined recognition of interspersed homologies in the repeat system that always included the 220-bp region of adjacent perfect homology, which was expected to provide a strong, persistent point of interaction. Those studies did not give any indication that RIP might be sensitive to the actual base-pair sequence of homologous units. In the present study, we have focused on the analysis of interspersed homologies spanning 500 base-pairs. We demonstrate that interspersed homologies with overall sequence identity of only 36% can be efficiently detected by RIP in the absence of perfect homology; yet short regions of adjacent perfect homology can have a strong activating effect. We have dissected the sequence requirements for RIP within one such short region of adjacent homology, under conditions where the overall amount of homology in the repeat system remains near the minimum threshold required for RIP. Analysis under these particularly stringent circumstances reveals that the base-pair composition of participating sequences can play a critical role in RIP and identifies one candidate trinucleotide, 5'-GAC-3', as an especially efficient unit of homology. We also present experimental evidence suggesting that RIP integrates sequence information over hundreds of base-pairs, and that the actual process of homology sensing can be distinguished from ensuing mutation. These and other results further illuminate the properties of DNA homology recognition during Neurospora RIP, paving the way for more mechanistic studies in the future.

## Results

### Background

**Fig 1:**
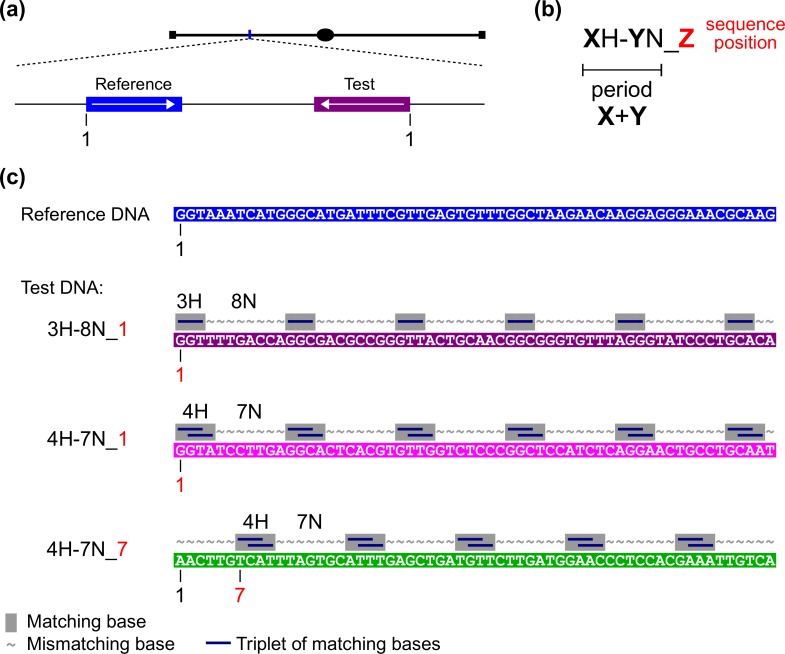
A sensitive genetic system for assaying RIP in regions of interspersed homology. (a) Tester constructs comprise pairs of closely-positioned DNA repeats: the base-pair sequence of the “left” repeat (“Reference”) is held constant and corresponds to an arbitrarily chosen segment of the Neurospora genome, the base-pair sequence of the “right” repeat (“Test”) can be varied as desired. (b) Patterns of interspersed homology (relative to the reference sequence) are defined by the three distances: **X**—the length of the homologous unit, **Y**—the separation between homologous units, and **Z**—the sequence position of the first homologous unit relative to the reference sequence. The sum of **X** and **Y** defines the periodicity of interspersed homology. (c) Alignment of a reference sequence and three different test sequences. A minimally effective pattern of interspersed homology is "3H-8N" [14]. One such pattern, with the sequence position parameter set to 1 (**Z** = 1), is shown as 3H-8N_1. Two homology patterns examined in this study are 4H-7N_1 and 4H-7N_7.

We previously developed a sensitive quantitative assay for RIP based on the analysis of individual mutations induced by a pair of closely-positioned DNA repeats [14]. The sequence of the “left” repeat (so-called "Reference") was always held constant, while the sequence of the “right” repeat (so-called "Test") was varied as desired (Fig 1A). To examine the capacity of different interspersed homologies to trigger RIP, we designed test sequences that contained short units of homology of length **X** separated by units of non-homology of length **Y**. The periodicity with which homologous units occurred along a particular pair of reference/test sequences is defined by the sum of these two distances (**X**+**Y**). Our previous research showed that only certain periodicities (**X**+**Y** = 11 and **X**+**Y** = 12) could promote RIP in conjunction with an adjacent region of perfect homology [14]. Because an array of homologous units can be positioned in a particular relationship with respect to the reference sequence, an additional parameter **Z** is now introduced to define the sequence position of the first homologous unit (Fig 1B). Patterns of interspersed homology investigated in this study are thus represented as **X**H-**Y**N_**Z** (Fig 1B). Because a particular reference region is selected arbitrarily from the Neurospora genome, the actual nucleotide sequences of homologous units are expected to be different for every combination of **X**, **Y**, and **Z**. However, by keeping **X** and **Y** constant and only changing the sequence position parameter **Z**, the sequences of homologous units can be altered without disrupting basic homology-block structure (Fig 1C). The present study shows that such sequence differences can be important for the efficiency of homology recognition for RIP.

### Short regions of perfect homology promote RIP mutation of long interspersed homologies

**Fig 2:**
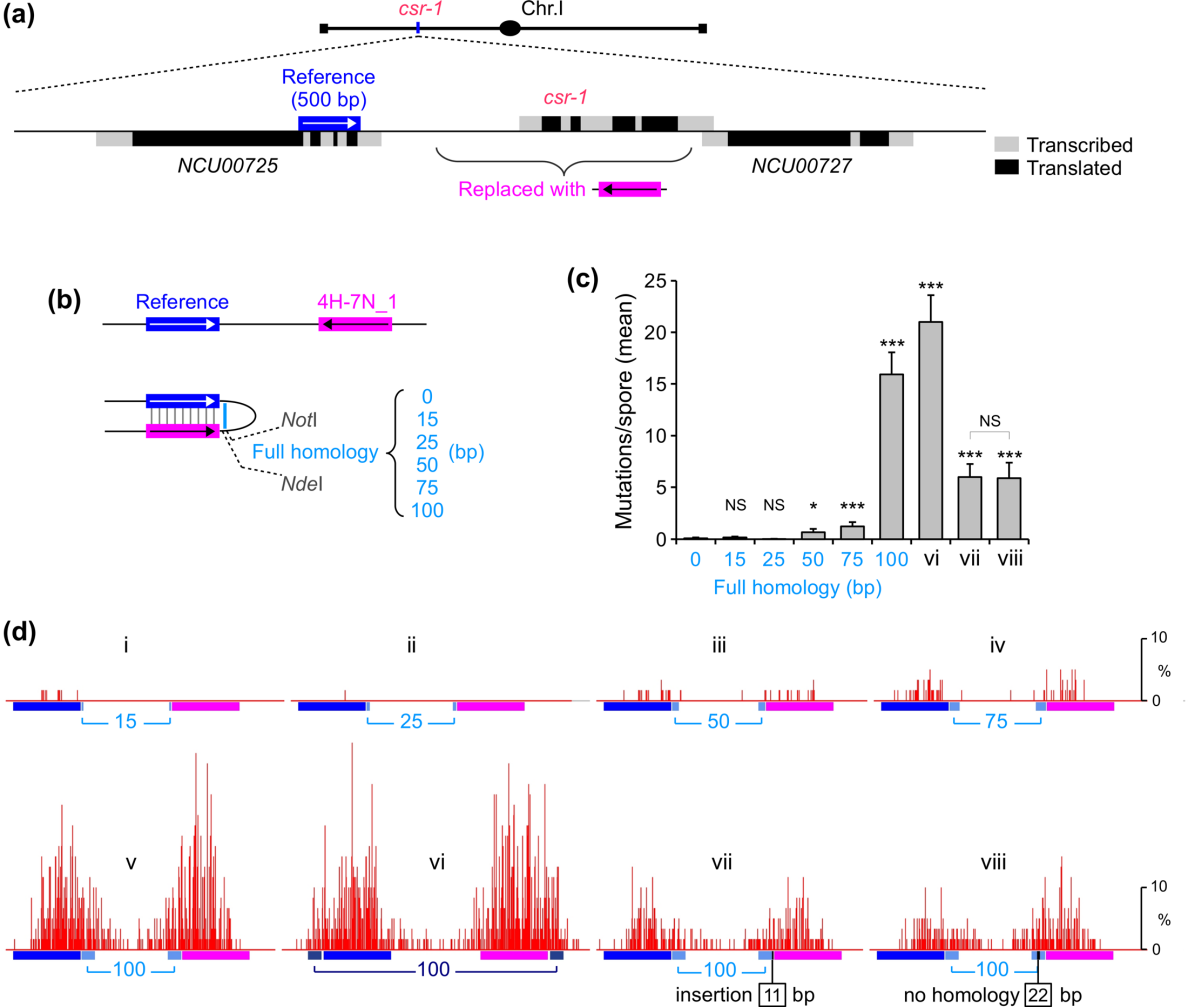
Short regions of perfect homology promote RIP mutation in combination with the 500-bp interspersed homology 4H-7N_1. (a) Partially-homologous repeats containing 500 base-pairs of the interspersed homology 4H-7N_1 (as defined in Fig 1A) are created by replacing the *cyclosporin-resistant-1* gene (*csr-1*) with synthetic DNA. (b) Derivatives of the basic repeat construct include 0–100 base-pairs of perfect homology created by integrating additional DNA between restriction sites *Not*I and *Nde*I. The repeat construct with 0 base-pairs of perfect homology was reported previously [14]. (c) The mean number of RIP mutations detected across the entire sequenced region for repeat constructs described in (Panels b and d). X-axis: Arabic numerals refer to the length of perfect homology inserted between *Not*I and *Nde*I sites, as shown in (Panel b); Roman numerals refer to additional repeat constructs tested in (Panel d). Error bars represent the standard error of the mean. The total number of sequenced spores and replica crosses are provided in S1 Table. (d) RIP mutation profiles for selected repeat constructs. Roman numerals correspond to the following RepeatIDs (provided in S1 Table): i—“XIQ”, ii—“XIO”, iii—“XIN”, iv—“XJG”, v—“XIM”, vi—“XKE”, vii—“XJH”, viii—“XJY”. The number of mutations per site is expressed as percent of all spores sequenced for a given repeat construct.

In our previous work, we analyzed mutation of the 500-bp interspersed homology 4H-7N_1 created by replacing the *cyclosporin-resistant-1* gene (*csr-1*) with synthetic DNA that was designed to be partially homologous to an arbitrarily selected reference region in a nearby gene (Fig 2A). In that study, we found that 4H-7N_1 alone exhibited barely detectable RIP but that, when a 337-bp region of perfect homology was present at an adjacent position, strong mutation of 4H-7N_1 was observed [14]. The present study was initiated to further define the role of perfect homology in promoting mutation of the interspersed homology 4H-7N_1. As in our previous analysis, the mean number of mutations (per spore) in the entire sequenced region was used as a quantitative measure of RIP. Empirical distributions of mutation counts were compared by the Kolmogorov-Smirnov test.

Systematic analysis shows that combining 4H-7N_1 with at least 50 base-pairs of perfect homology significantly increases RIP, and that 100 base-pairs of perfect homology promote very efficient RIP (Fig 2B–2D). A similar effect is observed regardless of whether the fully-homologous region is added to the "left" or the "right" end of the partially homologous region, implying that two different 100-bp sequences, at two different positions in the repeat construct, are equivalently effective (Fig 2D: compare v and vi). We further find that the insertion of 11 base-pairs of random DNA sequence at the junction between 4H-7N_1 and the fully-homologous region leads in a partial reduction in RIP, but that substantial mutation still occurs despite the apparent physical impediment to overall alignment (Fig 2D: compare v and vii). A similar effect is conferred by replacing 22 base-pairs of perfect homology by 22 base-pairs of non-homology in the middle of the 100-bp region, thereby interrupting the 100-bp perfect homology into two segments of 38 and 40 base pairs (Fig 2D: compare vii and viii).

**Fig 3:**
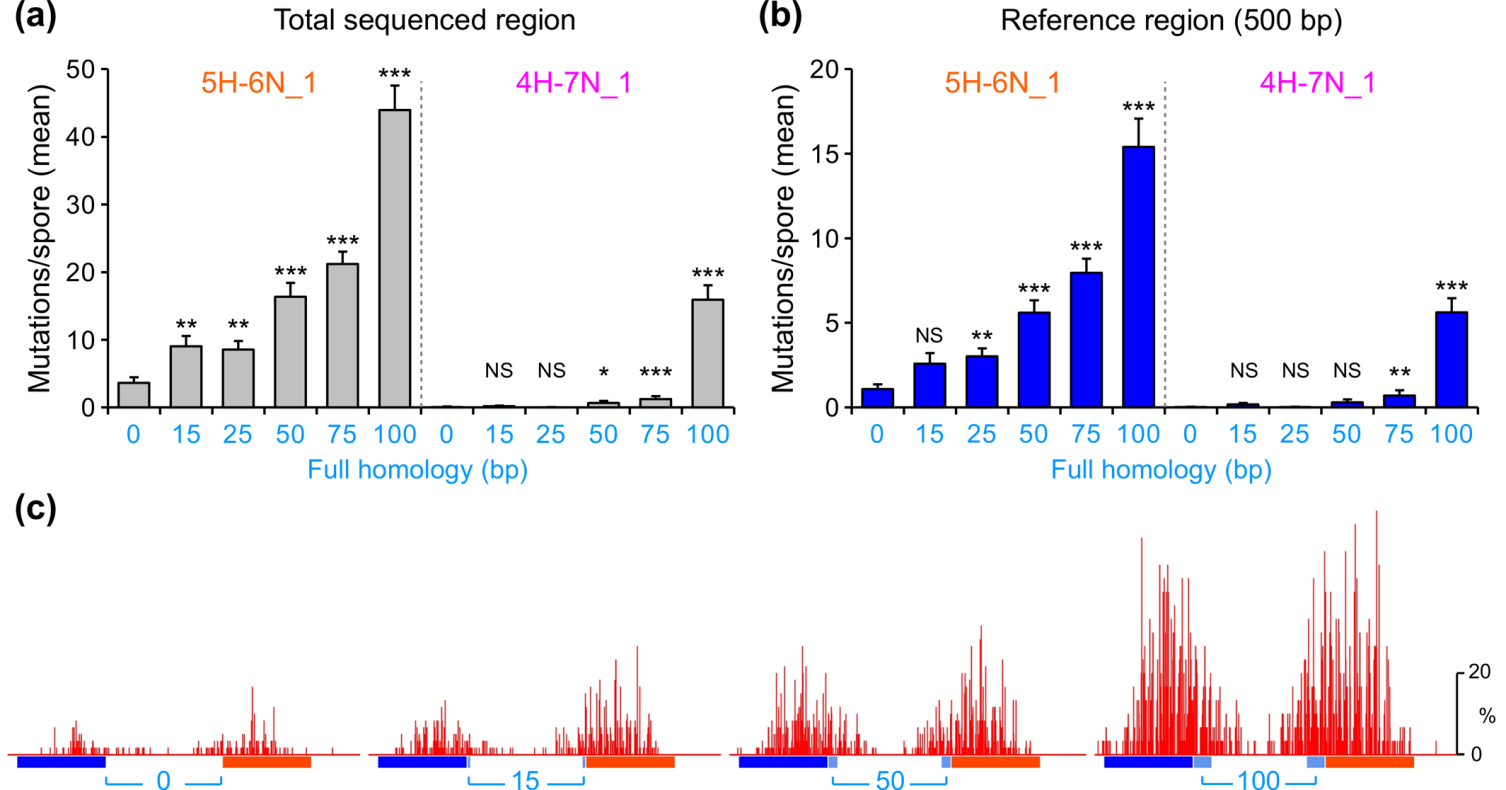
Short regions of perfect homology promote stronger mutation in combination with the 500-bp interspersed homology 5H-6N_1. (a) The mean number of RIP mutations (measured as in Fig 2C) for repeat constructs with 0–100 base-pairs of perfect homology adjacent to the interspersed homology 5H-6N_1 or 4H-7N_1. Data for the interspersed homology 4H-7N_1 are replotted from Fig 2C. (b) The mean number of RIP mutations detected across the 500-bp reference region for repeat constructs with 0–100 base-pairs of perfect homology adjacent to the interspersed homology 5H-6N_1 or 4H-7N_1. (c) RIP mutation profiles for selected repeat constructs containing 0, 15, 50 and 100 base-pairs of perfect homology adjacent to the interspersed homology 5H-6N_1.

We similarly examined the effect of adjacent perfect homology on mutation of another interspersed homology, 5H-6N_1 (Fig 3). This new homology pattern relates to the same reference sequence, and has the same periodicity (**X**+**Y**) and sequence position (**Z**) as 4H-7N_1, but contains homologous units of five base-pairs (**X** = 5) instead of four. We find that 5H-6N_1 alone can trigger substantial RIP, demonstrating a significant improvement over 4H-7N_1 (Fig 3). This observation provides a new conclusion: given an appropriate pattern of interspersed homology, perfect homology is dispensable for RIP.

We further find that, despite the substantial intrinsic activity of 5H-6N_1, the addition of only 15 base-pairs of perfect homology (representing 3% of the total homology length) increases RIP nearly 2.5-fold (from 3.6±0.9 to 9.0±1.5 mutations per spore, Fig 3A). This result has two implications. First, the presence of short perfect homology can still be highly advantageous even against the substantial background activity of 5H-6N_1. Second, such a strong response to 15 base-pairs of perfect homology contrasts with a negligible effect of combining the same 15-bp sequence with the interspersed homology 4H-7N_1 (Fig 3A and 3B). This difference might be explained by a synergistic interaction of the fully- and partially-homologous regions, such that more activity in the partially-homologous region leads to a greater effect of the fully-homologous region and *vice versa*. However, even from the more robust starting point of 5H-6N_1, and similarly to 4H-7N_1, a steep increase in mutation is observed when the region of perfect homology is extended from 75 base-pairs to 100 base-pairs, pointing to a critical threshold at about this length (Fig 3A and 3B). We also note that for all examined combinations of perfect and interspersed homologies, mutations tend to be distributed symmetrically near or around the center of each compound repeat, well inside the partially-homologous region. Taken together, the above findings further imply that the mechanism of homology recognition for RIP integrates sequence information from interspersed and perfect homologies collectively over several hundreds of base pairs.

### Interplay between the 500-bp interspersed homology 4H-7N_1 and different patterns of interspersed homology in the adjacent 100-bp region

**Fig 4:**
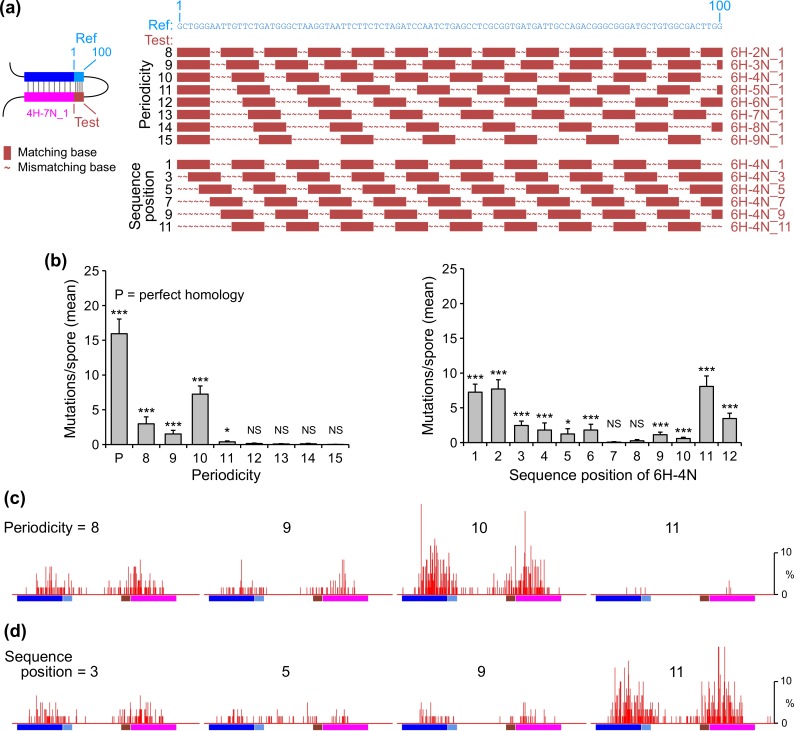
Interplay between the 500-bp interspersed homology 4H-7N_1 and different patterns of interspersed homology in the adjacent 100-bp region. (a) Reference/test combinations are created for a region derived from the 100-bp insert (Fig 2D: construct v). All interspersed homologies (as described in Fig 1) have a homologous unit length of 6 base-pairs and are varied with respect to periodicity (from 8 to 15) and sequence position (from 1 to 12, for the selected periodicity of 10). We note that the last homologous unit in 6H-4N_2, 6H-4N_3 and 6H-4N_4 was inadvertently extended by 3, 2 and 1 base-pairs, respectively. (b) The mean number of RIP mutations (measured as in Fig 2C) for repeat constructs described in (Panel a). (c) RIP mutation profiles for selected repeat constructs with periodicities of 8, 9, 10 and 11, corresponding to homology patterns 6H-2N_1, 6H-3N_1, 6H-4N_1 and 6H-5N_1, respectively, as described in (Panel a). (d) RIP mutation profiles for selected repeat constructs with sequence positions of 3, 5, 9, and 11, and corresponding to homology patterns 6H-4N_3, 6H-4N_5, 6H-4N_9 and 6H-4N_11, respectively, as described in (Panel a).

The above results show that recognition of long interspersed homologies can be promoted by relatively short segments of adjacent perfect homology. To obtain more insight into the interplay between these two homology types, we investigated the effects of substituting interspersed homology for perfect homology in a 100-bp region adjacent to 4H-7N_1 (Fig 2D, construct v). We chose to use 4H-7N_1 because it displayed almost no activity in the absence and strong activity in the presence of the adjacent 100-bp region (Fig 2C). We designed new patterns of interspersed homology in this 100-bp region by fixing the homologous unit length to 6 base-pairs (**X** = 6) and varying the other two parameters: periodicity (**X**+**Y**) and sequence position (**Z**) (Fig 4A). We have chosen **X** = 6 because our previous studies [14] suggested that homologous units of this particular length would provide a strong signal which, while not involving continuous homology, might have a significant effect on RIP in combination with 4H-7N_1. Systematic analysis shows that different patterns of adjacent interspersed homology can elicit different effects.

We first varied the periodicity parameter from 8 to 15 while keeping constant sequence position **Z** = 1. Corresponding interspersed homologies are shown in Fig 4A (from 6H-2N_1 to 6H-9N_1). A periodicity of 10 base-pairs confers the strongest effect, reaching nearly 50% the level observed with perfect homology; periodicities of 8 and 9 base-pairs induce lower but significant levels of RIP; and periodicities of 11–15 base-pairs produce little or no detectable effect (Fig 4B, left). These results were quite unexpected, because our previous analyses implied that periodicities of 11 or 12 should be favored [14], but are explained by further investigation (below).

We next explored the effects of varying the sequence-position parameter **Z**. In the above analysis, the 100-bp interspersed homology 6H-4N with sequence position **Z** = 1 (pattern 6H-4N_1) conferred the strongest positive effect on RIP. Thus, we have varied the sequence-position parameter of 6H-4N in this same context from **Z** = 1 to **Z** = 12 (Fig 4A). We find that sequence positions of 1 and 2 correspond to equally strong levels of RIP, with a progressive decrease for sequence positions 7–10, followed by an increase again for sequence positions 11 and 12 (Fig 4B, right). These results are intriguing: since the sequence-position parameter **Z** varies the nucleotide composition of the homologous units without altering basic homology-block structure, the possibility is raised that DNA sequence *per se* can have an important effect on RIP. In the context of this possibility, particular combinations of base-pairs in the homologous units of 6H-4N_1/11 and 6H-4N_2/12 might be optimal in synergizing with 4H-7N_1. However, alternatively, the differences in the ability to synergize, observed in this particular situation, could potentially arise from the variation in the distance of separation between homologous units at the junction between the 500-bp and 100-bp regions.

### RIP is modulated by the underlying DNA sequence

**Fig 5:**
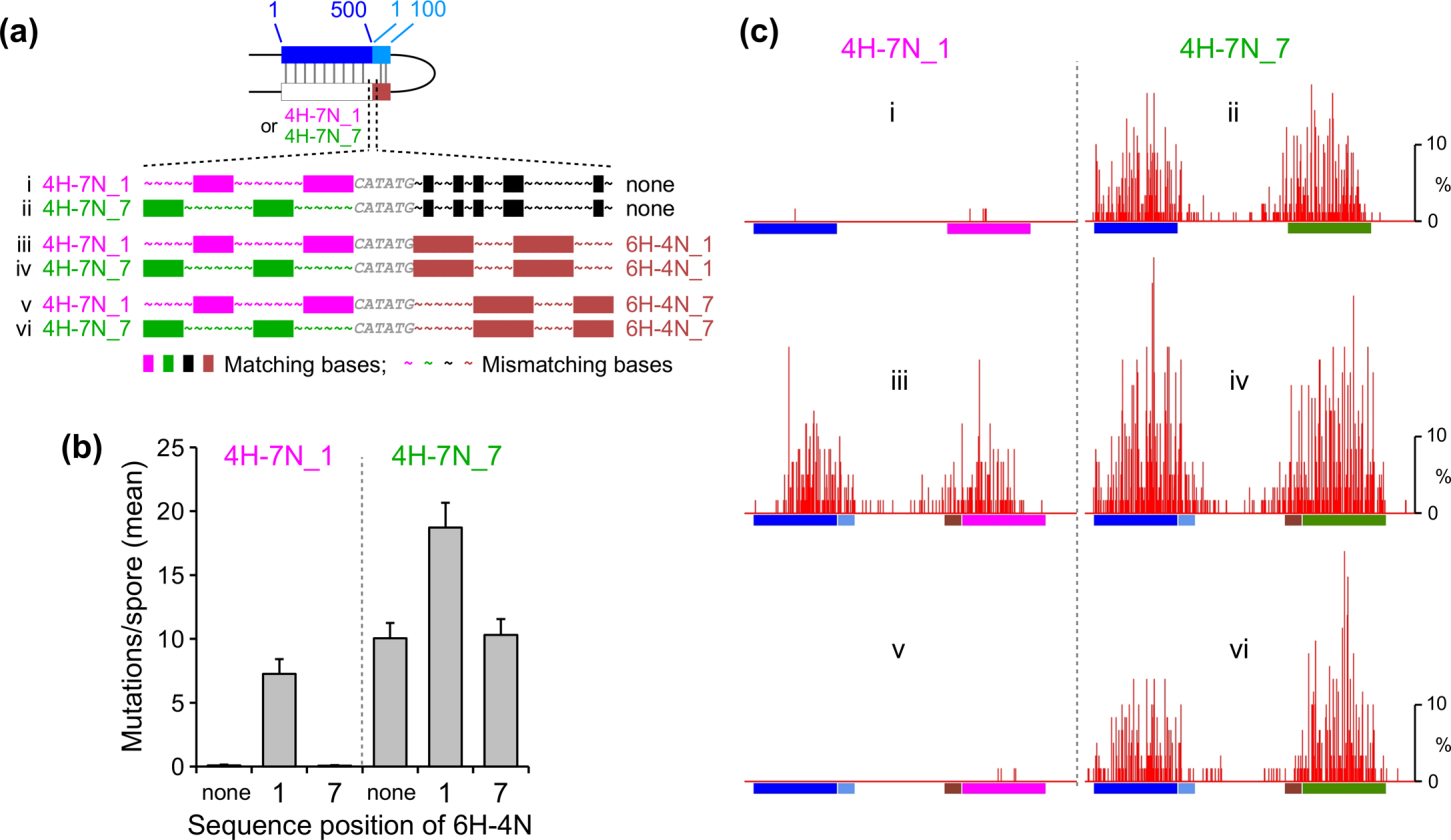
RIP is modulated by the underlying DNA sequence. (a) The 500-bp interspersed homologies 4H-7N_1 and 4H-7N_7 are tested in the absence of any adjacent homology (“none”, same as “0 bps”) or in combination with the 100-bp interspersed homologies 6H-4N_1 and 6H-4N_7. The last homologous unit in 4H-7N_1 was inadvertently extended by one base-pair. Roman numerals correspond to the following RepeatIDs (provided in S1 Table): i—“XIR” ii—“XKO”, iii—“XJJ”, iv—“XKM”, v—“XKC”, vi—“XKN”. (b) The mean number of RIP mutations (measured as in Fig 2C) for repeat constructs described in (Panel a). (c) RIP mutation profiles for repeat constructs described in (Panel a).

To further explore the issues raised in the previous section, we compared mutation of two 500-bp homologies that differed with respect to the sequence-position parameter: 4H-7N_1 (discussed above) and the newly designed interspersed homology 4H-7N_7 (Fig 5A, compare i and ii). We first examined these interspersed homologies alone, without any accompanying 100-bp region. Here we have observed a very dramatic difference with respect to their capacities to promote RIP (Fig 5B and 5C, compare i and ii): while RIP is virtually undetectable in the case of 4H-7N_1 (0.08±0.08 mutations per spore), it is quite strong in the case of 4H-7N_7 (10±1.2 mutations per spore). This comparison provides a clean demonstration that, in a situation where the overall level of homology is weak, differences in DNA sequence at positions of homology can have a substantial effect. In addition, these results show that periodically-spaced homologous units of four base-pairs, corresponding to overall sequence identity of only 36%, are sufficient to drive homology recognition.

We have then further examined the effects of the adjacent 100-bp regions 6H-4N_1 and 6H-4N_7 in combination with the 500-bp regions specifying 4H-7N_1 or 4H-7N_7 (Fig 5A). We find that each 100-bp region confers its characteristic effect, which appears superimposed on the effects conferred by the 500-bp regions: 6H-4N_1 increases RIP in both contexts, whereas 6H-4N_7 has no detectable effect in either context (Fig 5B and 5C). These results show that the relative sequence positions of homologous base-pairs within the 500-bp and 100-bp regions are not relevant—only the absolute sequence positions matter. These findings provide strong additional support for the notion that the sequence composition of homologous units is important for RIP.

### Identification of a specific triplet sequence that modulates RIP

**Fig 6:**
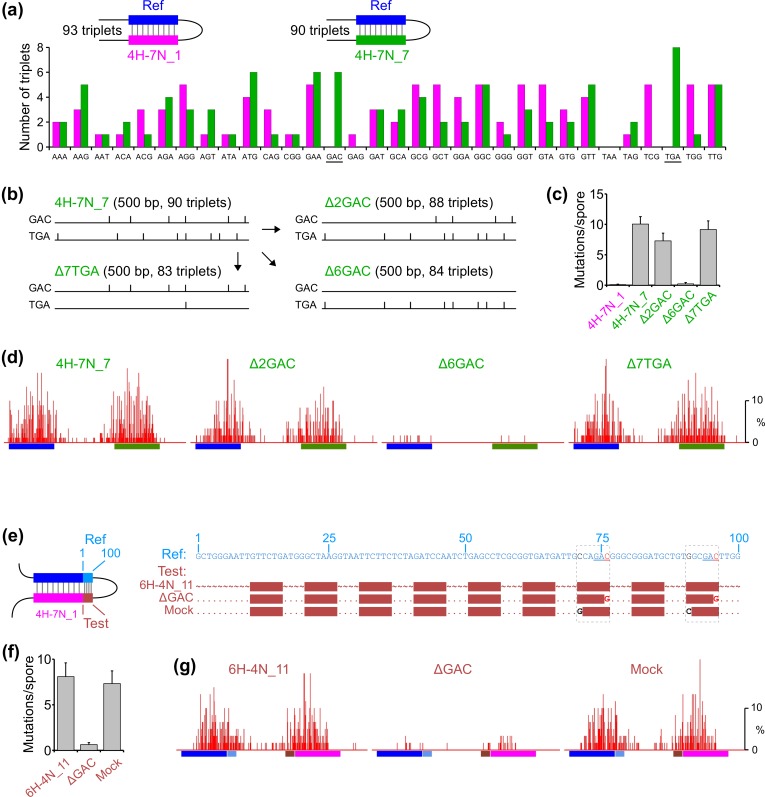
Identification of a specific triplet sequence that modulates RIP. (a) The number of triplets included in the homologous units of 4H-7N_1 and 4H-7N_7. Forward and reverse-complemented instances of each triplet are counted together. (b) Variants of the interspersed homology 4H-7N_7 that lack two GAC triplets (Δ2GAC), or all six GAC triplets (Δ6GAC), or seven TGA triplets (Δ7TGA). (c) The mean number of RIP mutations (measured as in Fig 2C) for repeat constructs described in (Panel b). (d) RIP mutation profiles for repeat constructs described in (Panel b). (e) The role of GAC triplets in the interspersed homology 6H-4N_11 was examined using the repeat construct described in Fig 4A. “ΔGAC”: both GAC triplets in the original sequence are deleted. "Mock": analogous mutations are made to delete two non-GAC triplets, as described in the text. (f) The mean number of RIP mutations (measured as in Fig 2C) for repeat constructs in (Panel e). (g) RIP mutation profiles for repeat constructs described in (Panel e).

If DNA sequence plays a critical role in RIP, what is the basis for this effect? Our previous work has suggested that the triplet of base-pairs represents the basic unit of homology recognition for RIP [14]. Therefore, we wondered whether, among other sequence determinants, “RIP-proficient” homologies might have a higher abundance of certain triplets as compared to “RIP-deficient” homologies. We have shown that the 500-bp homologies 4H-7N_1 and 4H-7N_7, which differ in the positions of the homologous units and, therefore, have the homologous units comprising different base-pair combinations, exhibit a 100-fold difference in their ability to promote RIP. Thus, we have compared nucleotide sequences of 4H-7N_1 and 4H-7N_7 with respect to the natures of triplets included in their homologous units (Fig 6A). We find that most triplets are present in comparable numbers, but with two exceptions: six GAC and eight TGA triplets are present in 4H-7N_7 (which promotes substantial RIP), while neither of these triplets appears in 4H-7N_1 (which promotes very little RIP).

To investigate whether either or both of these differences were significant for RIP, we first explored the role of GAC triplets. We have designed two variants of 4H-7N_7 that lacked either two or all six GAC triplets (Fig 6B, patterns “Δ2GAC” and “Δ6GAC”). The single-nucleotide substitutions that specifically eliminated GAC triplets were chosen to accommodate the fact that each 4-bp unit of homology in 4H-7N_7 contains two overlapping triplets (Fig 1C), making it possible to alter the GAC triplet without affecting the other overlapping triplet. This is accomplished by mutating G to C if the GAC triplet occupies positions 1–3 of the 4-bp unit, or by mutating C to G if the GAC triplet occupies positions 2–4. Our results show that removing the two GAC triplets has reduced RIP by about 25%, while removing all six GAC triplets nearly eliminated all RIP activity (Fig 6C and 6D).

We next analogously examined the possible role of TGA triplets (Fig 6B, pattern “Δ7TGA”). In contrast to the results obtained for GAC triplets, deleting seven TGA triplets using the above approach had no significant effect on RIP (Fig 6C and 6D). Taken together, these results strongly suggest that GAC triplets, but not TGA triplets, may play a privileged role in RIP.

As an additional confirmation of this conclusion, we have examined the role of GAC triplets in another context. Our results have shown that the 100-bp region of perfect homology produces a much stronger increase in mutation compared to the 75-bp region (Fig 3A). We have noticed that the 100-bp region, but not the 75-bp region, contains two GAC triplets (Fig 6E). These GAC triplets are also present in the 100-bp interspersed homology 6H-4N_11 that promotes substantial RIP in combination with 4H-7N_1 (Fig 4B, right). Using the same approach as described above, we mutated the two GAC triplets in 6H-4N_11 (Fig 6E: “ΔGAC”). As a control, we introduced two C/G and G/C substitutions in unrelated triplet*s* (Fig 6E: “Mock”). Mutation analysis shows that the two single-nucleotide changes that deleted the GAC triplets have effectively eliminated the ability of 6H-4N_11 to activate RIP, while the two changes that removed non-GAC triplets have had no significant effect (Fig 6F and 6G). These findings further support the idea that GAC triplets may indeed have a special role in RIP.

### Recognition of homology and ensuing mutation are not coupled with respect to the involved base-pairs

**Fig 7:**
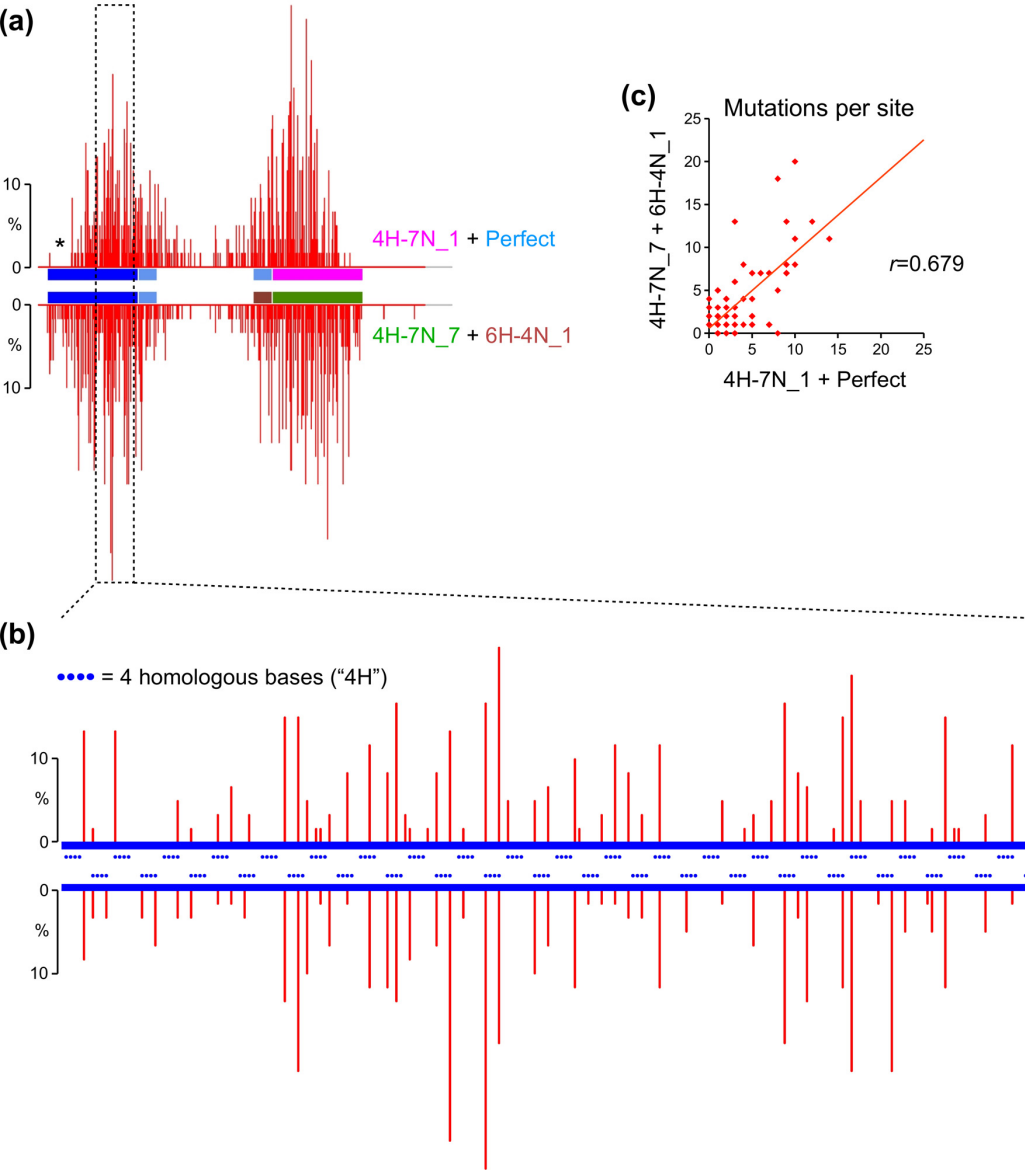
Homology recognition and ensuing mutation are not coupled with respect to the involved base-pairs. (a) RIP mutation profiles of the two diagrammed repeat constructs are compared along their entire lengths. A 200-bp portion of the reference sequence that appears to have similar levels of RIP in both cases is outlined. We note that a distal portion of the same reference sequence (marked with “*”) escapes RIP in the case of 4H-7N_1 but not in the case of 4H-7N_7. (b) RIP mutation profiles along the 200-bp portion of the reference sequence at the base-pair resolution. “●●●●” indicates the positions, in the reference sequence, of the 4-bp homologous units defined by the corresponding test sequences 4H-7N_1 (upper profile) and 4H-7N_7 (lower profile). (c) Correlation of mutations observed for individual sites within the 200-bp segment (Panel b). *r* is the Pearson correlation coefficient.

Previous research had shown that repeat units were mutated specifically over the extent of shared homology, with only a few mutations occurring outside the duplicated regions [16]. Such high accuracy implies tight coupling between the processes of homology recognition and ensuing mutation. However, our previous work showed that single-copy regions located between pairs of closely-positioned repeats could be strongly mutated, suggesting that homology recognition and mutation can, in fact, be uncoupled regionally [14]. Here we explored the possibility of uncoupling at the base-pair level. Specifically, we have asked whether the local occurrence of mutations is affected by the positions of homologous units. For this purpose, we have compared mutation patterns produced by interspersed homologies 4H-7N_1 and 4H-7N_7 in combination with appropriate 100-bp regions: (i) 4H-7N_1 with 100 base-pairs of perfect homology and (ii) 4H-7N_7 with 100 base-pairs of the interspersed homology 6H-4N_1 (Fig 7A). These repeat constructs share the same reference sequence, but, because of the difference in the sequence-position parameter, their 4-bp homologous units do not overlap (Fig 7B). While the overall RIP profiles appear somewhat different between the two repeat constructs, with the apparent paucity of mutations in the first ~130 base-pairs of 4H-7N_1 (Fig 7A, marked with “*”), similar levels of RIP can be observed within the 200-bp portion of the reference sequence (Fig 7A, outlined). Focusing our analysis on this 200-bp region, we find that cytosines at identical positions are mutated similarly and irrespectively of their spatial relationship to the underlying homologous units (Fig 7B and 7C). This result suggests that, at the level of individual base-pairs, homology recognition and mutation are separable events, either functionally and/or temporally.

## Discussion

We previously examined recognition of interspersed homologies using repeat constructs that included a 200-bp region of tested interspersed homology adjoining a 220-bp region of perfect homology [14], see also (S1 Fig). In this context, the effective patterns of interspersed homology collectively implied that the sequence information was sensed in units of three base-pairs spaced at intervals of 11 or 12 base-pairs, over the total length of several hundred base-pairs. We interpreted this result as evidence that two co-aligned double-stranded DNA molecules were compared by direct contacts. The current study extends these findings in several respects.

- First, we show that interspersed homologies can be efficiently recognized by RIP in the absence of any adjacent perfect homology.
- Second, we show that short regions of perfect homology can still dramatically increase RIP in the adjacent region of interspersed homology, where stronger activity of the partially-homologous region leads to a greater effect of the fully-homologous region and *vice versa*. These results point to the synergistic interplay among homologous sequences along the same DNA segment and support the idea that the mechanism of homology recognition for RIP integrates sequence information over several hundred base-pairs.
- Third, we have explored, for the first time, the possible role of the underlying DNA sequence in homology recognition for RIP. For this purpose, we compared mutation patterns triggered by different interspersed homologies with the same unit length and the same periodicity but with different base-pair compositions of homologous units as dictated by their sequence positions along the same reference region. We find that the base-pair composition in homologous units can play a critical role in RIP and identify the triplet 5'-GAC-3' as a particularly favorable unit of homology. The latter result provides further support for the idea that homology is being sensed in groups of base-pairs, specifically, in triplets. We note, however, that interspersed homologies lacking GAC triplets (e.g., 5H-6N_1, Fig 3) can still promote substantial RIP, suggesting that GAC triplet(s) are not essential. Moreover, short interspersed homologies with the same GAC triplets (e.g., 6H-4N_2 and 6H-4N_12, Fig 4) appear to promote RIP at significantly different levels, implying that other factor(s), e.g. the sequence composition of non-homologous units, can have an influence.
- Finally, our new results provide strong evidence that the analyses of sequence requirements in our current and previous studies are actually examining the process of homology recognition *per se*, and not its downstream effect on mutation. Specifically, we show that the same cytosines in a given reference sequence are mutated with similar efficiencies, regardless of which particular base-pairs of that sequence are involved in homologous interactions with corresponding test sequences comprising different patterns of interspersed homology. This finding concomitantly implies that RIP proceeds in two separable steps of (i) defining the overall homology and then (ii) modifying the underlying sequences.

Taken together, previous and current findings make it clear that a number of different sequence features contribute to DNA homology recognition for RIP. Our previous study [14] identified triplet homology units and 11/12 base-pair periodicities of those units as important features for RIP. The present study uncovers another important factor: the underlying DNA sequence in general and GAC triplets in particular. It is important to appreciate that different features emerged in the two studies because of the built-in differences in the repeat configurations being used. In the previous work, we examined variations in the basic pattern of interspersed homology within a 200-bp region that was positioned adjacent to a 220-bp region of perfect homology which, by itself, triggered substantial RIP. Thus, the effectiveness of different homology patterns was being evaluated in a situation where the basal level of homology (provided by the 220-bp region) was already above the critical threshold required for RIP. In this experimental system, a preference for homology in triplets of base-pairs spaced with the 11- or 12-bp periodicity was revealed. The present study, in contrast, examined situations in which the basal level of homology was close to or below the critical threshold for RIP. Starting with the intrinsically weak interspersed homology 4H-7N_1 (Fig 2), we found that 100 base-pairs of adjacent perfect homology promoted strong RIP, whereas 75 base-pairs of perfect homology were much less effective, implying that the 100 base-pairs were just barely effective. Starting from this suboptimal situation, examining requirements for interspersed homology in the 100-bp region revealed a role of the underlying DNA sequence. The actual patterns of interspersed homology that permitted the strongest effect did not obviously involve 11/12-bp periodicities. This is likely because the 100-bp region is predicted to contain only ~9 duplex/duplex contact points rather than ~18 as in the previous study, thus giving the DNA sequence a more prominent role. Overall, these observations highlight the fact that homology recognition for RIP can collectively integrate and evaluate diverse underlying features over substantial distances.

Evidence that RIP involves direct dsDNA/dsDNA homology recognition is still indirect. In principle, homology recognition might involve dsRNA interacting with dsDNA or, even, some other type of sequence-specific interactions not involving two double-helical nucleic acids. We note, however, that RIP can recognize 4-bp units of homology embedded in a completely non-homologous sequence, and that four base-pairs are significantly below the threshold length of 6–7 nucleotides required for stable pairing of single-stranded nucleic acids in the context of Argonaute proteins [17], arguing against the role of these proteins in homology sensing for RIP.

The presented observations provide new grounds to justify consideration of existing models of sequence-specific pairing between intact DNA molecules. Two long-standing models invoke the intriguing principle of self-complementarity of Watson-Crick base-pairs, by which identical base-pairs can form planar quadruplexes *via* major-groove [18] or minor-groove [19] interactions. The possibility of such interactions was confirmed experimentally by NMR [20,21]. Further, in response to our previous work, the principle of base-pair self-complementarity was suggested to be capable of mediating pairing between long intact DNA molecules by the formation of short interspersed dsDNA/dsDNA quadruplexes [22]. This model also predicts that such quadruplex interactions may be sensitive to the underlying DNA sequence [22]. However, pairing of long double-stranded DNAs by this mechanism could involve two duplexes that are either plectonemically intertwined over the "pairing region" (which would permit many contacts along the region) or paranemically related (which would limit direct contacts to one per ~55 base-pairs) [22]. Neither of these conditions is impossible but also neither is necessarily attractive *a priori*.

Another proposed model includes the intertwining of two negatively supercoiled double-stranded DNA molecules in a form of so-called “PX-DNA”, where homology recognition is mediated by standard Watson-Crick hydrogen bonds [23]. Recent data, however, suggest that the actual structure of the paired complex in this case remains elusive [24]. In a third type of model, non-Watson-Crick hydrogen bonds are proposed to mediate association of specialized sequences, such as G-quartets [25] or triplex structures involving long polypurine/polypyrimidine runs [26]. However, the RIP phenomenon in general, and the newly discovered patterns of sequence recognition in particular, are not compatible with such specialized mechanisms.

Finally, interactions between homologous DNA molecules were also proposed to occur in the absence of direct contacts, by a very different type of a mechanism called "electrostatic zipper" [27]. In this model, two double-stranded DNA molecules with identical sequences can align by making multiple electrostatic contacts between negatively charged backbone phosphates on one duplex and positive charges in the grooves of the other duplex. In this model, DNA homology is read out indirectly: pairing occurs specifically between homologous sequences because only such sequences can produce complimentary patterns of negative and positive charges [27]. As pointed out by the authors, this model cannot account energetically for pairing between DNA molecules that are significantly different as is observed for the interspersed homologies that trigger RIP [28].

Consideration of these models, together with our constraining results, suggests that direct DNA/DNA homology recognition, which also occurs *in vitro* [29] may involve mechanism(s) that still remain(s) to be described. Furthermore, regardless of the local basis for homology recognition, there also remains the fundamental unsolved issue of how a pair of relatively short chromosomal regions with similar nucleotide sequences can accurately identify one another within the vast space and genomic complexity of the nucleus on a time scale that makes the “genome-by-genome” homology search for RIP feasible.

In Neurospora, RIP evolved as a genome defense mechanism to control the expansion of mobile DNA [15]. In most eukaryotic organisms, including mammals, genomes contain vast amounts of repetitive DNA normally silenced in the form of heterochromatin [30]. While the role of RNA intermediates and sequence-specific DNA binding proteins were clearly implicated in many cases involving epigenetic silencing of DNA repeats [31,32], in the other cases there is no clear understanding how the heterochromatic state can be induced over repetitive sequences [33,34,35]. It is possible that homology-dependent interactions analogous to those that underlie RIP could play a not yet considered role in other phenomena that promote the assembly of heterochromatin on repetitive DNA. We also note that recombination-independent pairing is a prominent feature of inter-chromosomal interactions in both somatic and meiotic systems [2], and thus the rules that underlie homology recognition for RIP may well underlie a wide variety of homology-dependent phenomena in other biological systems.

## Materials and Methods

Methods for creating interspersed homologies, constructing plasmids and strains, setting up crosses, recovering RIP products and analyzing mutations were previously described [14]. Briefly, interspersed homologies were designed *in silico* by substituting each designated base with one of the three remaining alternatives chosen with equal probabilities and independently from neighboring bases. The exact algorithm for creating interspersed homologies (written in Perl) is provided in S4 File. Synthetic DNA was ordered as “gBlocks” from Integrated DNA Technologies. Repeat cassettes were integrated as a replacement of the *csr-1* gene in the *mus-52*Δ strain FGSC#9720, which is deficient in the non-homologous end joining pathway and can only be transformed by homologous recombination [36]. 1–2 homokaryotic transformants were typically selected for further analysis. All integration events were validated by sequencing. Plasmids and strains created in this study are listed in S1 Table. Individual plasmid maps (in GenBank format) are provided in S1 File (as tar/gz archive).

The standard wildtype strain FGSC#4200 was used as a female parent for all the crosses. 1–3 replica crosses were analyzed for each repeat construct; at least 30 random “late” spores were randomly sampled from each cross. The number of replica crosses and the total number of analyzed spores are provided in S1 Table. PCR-amplified repeat cassettes were sequenced directly by the Sanger method. Sequencing reactions were read with an ABI3730xl DNA analyzer at the DNA Resource Core of Dana-Farber/Harvard Cancer Center (funded in part by NCI Cancer Center support grant 2P30CA006516-48). Individual chromatograms were assembled into contigs with Phred/Phrap. Assembled contigs were inspected manually in Consed. Sequences of all contigs analyzed in this study are provided in S3 File.

## Supporting Information

S1 Fig Analysis of 200-bp interspersed homologies adjacent to the 220-bp region of perfect homology, as described in [14]. (a) The basic repeat construct. (b) All triplets that occur within the 220-bp region of perfect homology (analyzed as in Fig 6A). (c) All triplets that occur in the “3H” units of interspersed homologies examined in [14]. “RIP-proficient” interspersed homologies are outlined in dashed red.
(PDF)

S1 Table Repeat constructs analyzed in this study. **Construct**, unique description of each repeat construct; **RepeatID**, unique identifier of each repeat construct; **Plasmid**, unique identifier of each repeat-carrying plasmid (plasmid maps are provided in S1 File); **Strain(s)**, unique identifiers of repeat-carrying strains produced by transforming the recipient strain FGSC#9270 with a linearized plasmid; **N(X)**, the number of replica crosses; **P (min)**, the lowest P-value obtained by the Kolmogorov-Smirnov test of congruence between all possible pairs of replica crosses; **N(S)**, the total number of spores analyzed for each repeat construct; **Mean**, the mean number of RIP mutations (per spore) identified for each repeat construct; **SEM**, standard error of the mean.
(PDF)

S1 File Plasmids created in this study. Plasmid maps are in the GenBank Flat File Format. Individual files are provided as a single tar/gzip archive. Plasmid names are listed in S1 Table.
(GZ) Click here for additional data file.

S2 File Patterns of homology analyzed in this study. Pairwise alignments of reference/test sequences (left/right) for each repeat construct. Individual ClustalW files are provided as a single tar/gzip archive. Alignment file names correspond to RepeatID in S1 Table.
(GZ) Click here for additional data file.

S3 File Complete sequences of all assembled contigs analyzed in this study. Sequences are provided in the comma-separated (CSV) format. The file is compressed with gzip. The dataset contains four fields: [1] Unique sequence identifier, [2] the total number of C/T mutations, [3] the total number of G/A mutations, and [4] the complete nucleotide sequence. Sequence identifiers are provided in the following format: {RepeatID}{Replica Cross Index}_{Spore Index} (e.g., notation “{XIF}{1}_{1}” corresponds to the actual sequence identifier “XIF1_1”). “XIR” sequences were published previously [14] and are provided here for convenience.
(GZ) Click here for additional data file.

S4 File The algorithm for generating interspersed homologies. The algorithm is written in Perl.and requires BioPerl. The script is provided as a tar/gzip archive.
(GZ) Click here for additional data file.
