# Supplementary material for: Recombination-Independent Recognition of DNA Homology for Repeat-Induced Point Mutation (RIP) Is Modulated by the Underlying Nucleotide Sequence
Source: PLoS Genet. 2016 May 5;12(5):e1006015. doi: 10.1371/journal.pgen.1006015 (PMC4858203; doi:10.1371/journal.pgen.1006015)
Supplement: S1 Fig — (a) The basic repeat construct. (b) All triplets that occur within the 220-bp region of perfect homology (analyzed as in Fig 6A). (c) All triplets that occur in the “3H” units of interspersed homologies examined in [14]. “RIP-proficient” interspersed homologies are outlined in dashed red. (PDF) [file pgen.1006015.s001.pdf]

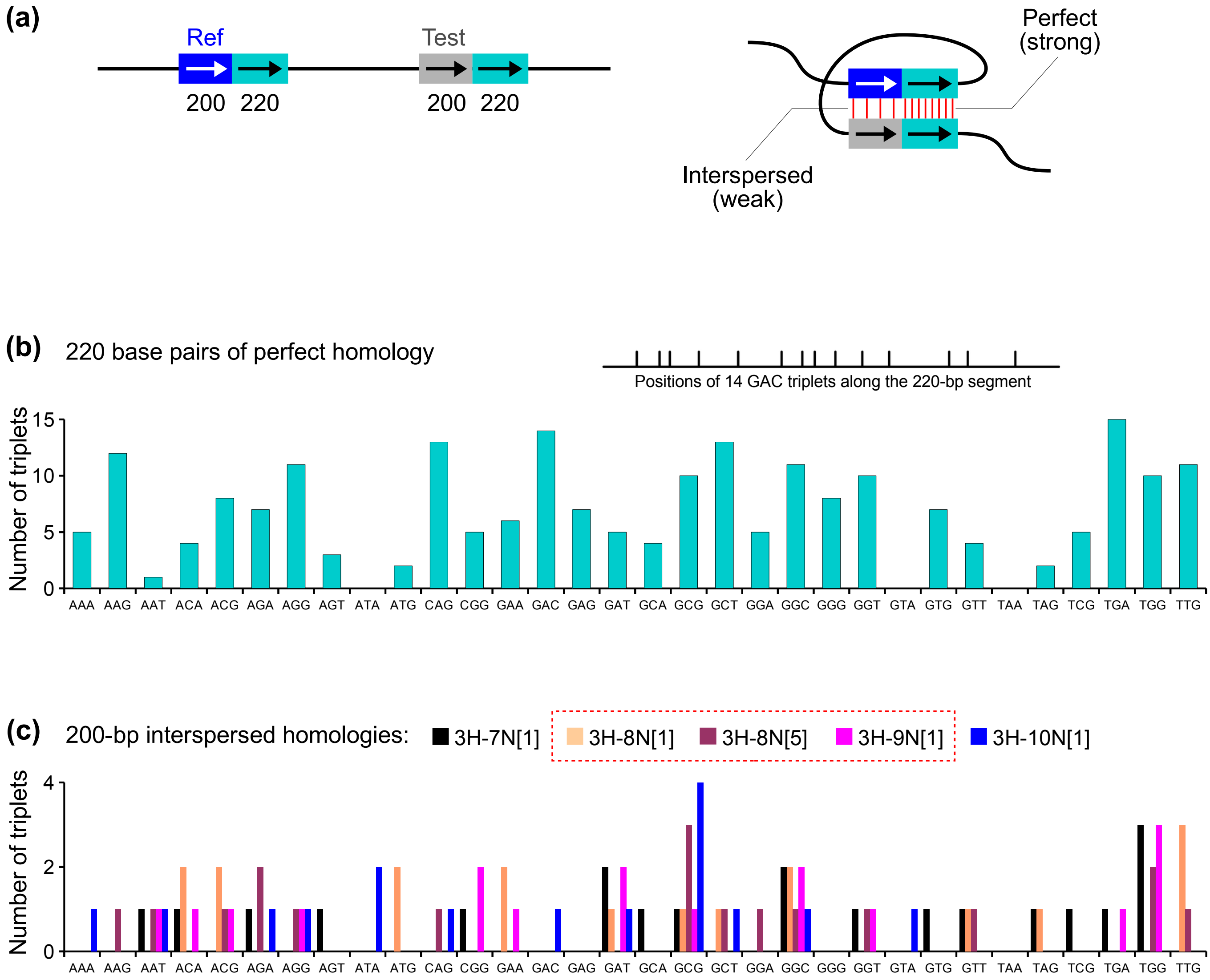

S1 Fig. Analysis of 200-bp interspersed homologies adjacent to the 220-bp region of perfect homology, as described in [14]
(a) The basic repeat construct.
(b) All possible triplets that occur within the 220-bp region of perfect homology (analyzed as in Fig 6a).
(c) All possible triplets that occur in the "3H" units of interspersed homologies examined in [14]. "RIP-proficient" interspersed homologies are outlined in dashed red.
