# Supplementary material for: Recombination-Independent Recognition of DNA Homology for Repeat-Induced Point Mutation (RIP) Is Modulated by the Underlying Nucleotide Sequence
Source: PLoS Genet. 2016 May 5;12(5):e1006015. doi: 10.1371/journal.pgen.1006015 (PMC4858203; doi:10.1371/journal.pgen.1006015)
Supplement: S1 Table — Construct, unique description of each repeat construct; RepeatID, unique identifier of each repeat construct; Plasmid, unique identifier of each repeat-carrying plasmid (plasmid maps are provided in S1 File); Strain(s), unique identifiers of repeat-carrying strains produced by transforming the recipient strain FGSC#9270 with a linearized plasmid; N(X), the number of replica crosses; P (min), the lowest P-value obtained by the Kolmogorov-Smirnov test of congruence between all possible pairs of replica crosses; N(S), the total number of spores analyzed for each repeat construct; Mean, the mean number of RIP mutations (per spore) identified for each repeat construct; SEM, standard error of the mean. (PDF) [file pgen.1006015.s002.pdf]

S1 Table. Repeat constructs analyzed in this study

Construct	RepeatID	Plasmid	Strain(s)	N(X)	P (min)	N(S)	Mean	SEM
4H-7N_1 + 100 bps	XIM	pEAG199M	T306.2	2	0.05	60	15.93	2.13
4H-7N_1 + 75 bps	XJG	pEAG204G	T336.1; T336.3	2	0.12	60	1.23	0.43
4H-7N_1 + 50 bps	XIN	pEAG199N	T287.8	2	0.30	60	0.67	0.32
4H-7N_1 + 25 bps	XIO	pEAG199O	T288.1; T288.3	2	1.00	60	0.02	0.02
4H-7N_1 + 15 bps	XIQ	pEAG199Q	T290.4	2	0.24	60	0.17	0.10
4H-7N_1 + 0 bps	XIR	pEAG199R	T293.2; T293.4	2	1.00	60	0.08	0.08
4H-7N_1 + 100 bps {outside}	XKE	pEAG223E	T387.9	1	N/A	60	21.00	2.60
4H-7N_1 + {11 ins} + 100 bps	XJH	pEAG204H	T338.4	2	1.00	60	5.98	1.27
4H-7N_1 + 100 {22N} bps	XJY	pEAG204Y	T369.12; T369.14	2	0.05	60	5.87	1.52
5H-6N_1 + 100 bps	XIF	pEAG199F	T278.3	1	N/A	30	43.93	3.64
5H-6N_1 + 75 bps	XJF	pEAG204F	T335.8; T335.9	2	1.00	60	21.18	1.83
5H-6N_1 + 50 bps	XIG	pEAG199G	T274.6	2	0.14	60	16.37	2.02
5H-6N_1 + 25 bps	XIH	pEAG199H	T275.1	2	0.38	60	8.57	1.23
5H-6N_1 + 15 bps	XIL	pEAG199L	T286.3; T286.5	2	0.99	60	9.02	1.53
5H-6N_1 + 0 bps	XII	pEAG199I	T276.1; T276.2	2	0.86	60	3.63	0.85
4H-7N_1 + 6H-2N_1	XJP	pEAG204P	T361.2; T361.4	2	0.35	60	2.97	1.00
4H-7N_1 + 6H-3N_1	XJQ	pEAG204Q	T360.3	2	0.70	60	1.52	0.51
4H-7N_1 + 6H-5N_1	XJK	pEAG204K	T341.4; T341.6	2	0.85	60	0.37	0.15
4H-7N_1 + 6H-6N_1	XJL	pEAG204L	T342.1; T342.3	2	0.62	60	0.13	0.07
4H-7N_1 + 6H-7N_1	XJM	pEAG204M	T343.1; T343.2	2	1.00	60	0.07	0.04
4H-7N_1 + 6H-8N_1	XJN	pEAG204N	T350.5; T350.7	2	0.61	60	0.10	0.06
4H-7N_1 + 6H-9N_1	XJO	pEAG204O	T344.3; T344.4	2	1.00	60	0.02	0.02
4H-7N_1 + 6H-4N_1	XJJ	pEAG204J	T340.1; T340.2	2	0.43	60	7.25	1.16
4H-7N_1 + 6H-4N_2	XKA	pEAG223A	T383.2; T383.4	2	0.54	60	7.70	1.33
4H-7N_1 + 6H-4N_3	XKB	pEAG223B	T384.10; T384.9	2	0.82	60	2.45	0.64
4H-7N_1 + 6H-4N_4	XKF	pEAG223F	T393.1	1	N/A	30	1.80	1.03
4H-7N_1 + 6H-4N_5	XJT	pEAG204T	T365.15; T365.3	2	0.80	60	1.23	0.77
4H-7N_1 + 6H-4N_6	XKG	pEAG223G	T394.4	1	N/A	30	1.80	0.83
4H-7N_1 + 6H-4N_7	XKC	pEAG223C	T385.19	2	1.00	60	0.07	0.05
4H-7N_1 + 6H-4N_8	XKH	pEAG223H	T399.2	2	0.24	60	0.25	0.19
4H-7N_1 + 6H-4N_9	XKD	pEAG223D	T386.7; T386.9	2	0.74	60	1.12	0.38
4H-7N_1 + 6H-4N_10	XKI	pEAG223I	T395.3; T395.4	2	0.79	60	0.58	0.17
4H-7N_1 + 6H-4N_11	XKJ	pEAG223J	T396.1	2	0.73	60	8.08	1.51
4H-7N_1 + 6H-4N_12	XKK	pEAG223K	T397.2	2	0.92	60	3.45	0.78
4H-7N_7 + 0 bps	XKO	pEAG223O	T419.11; T419.25	3	0.76	90	10.04	1.22
4H-7N_7 + 6H-4N_1	XKM	pEAG223M	T400.2	2	0.55	60	18.72	1.94
4H-7N_7 + 6H-4N_7	XKN	pEAG223N	T401.1	2	0.62	60	10.32	1.23
4H-7N_7 {Δ2GAC} + 0 bps	XKW	pEAG223W	T455.1	1	N/A	60	7.28	1.27
4H-7N_7 {Δ6GAC} + 0 bps	XKP	pEAG223P	T431.2; T431.3	2	1.00	60	0.22	0.22
4H-7N_7 {Δ7TGA} + 0 bps	XKZ	pEAG223Z	T450.3; T450.4	2	0.82	60	9.15	1.42
4H-7N_1 + 6H-4N_11{dGAC}	XKX	pEAG223X	T433.6; T433.7	2	1.00	60	0.63	0.23
4H-7N_1 + 6H-4N_11{Mock}	XKY	pEAG223Y	T434.1; T434.5	2	0.50	60	7.33	1.39

Construct, unique description of each repeat construct; **RepeatID**, unique identifier of each repeat construct; **Plasmid**, unique identifier of each repeat-carrying plasmid (plasmid maps are provided in S1 File); **Strain(s)**, unique identifiers of repeat-carrying strains produced by transforming the recipient strain FGSC#9270 with a linearized plasmid; **N(X)**, the number of replica crosses; **P (min)**, the lowest P-value obtained by the Kolmogorov-Smirnov test of congruence between all possible pairs of replica crosses; **N(S)**, the total number of spores analyzed for each repeat construct; **Mean**, the mean number of RIP mutations (per spore) identified for each repeat construct; **SEM**, standard error of the mean.
